# Evolutionary adaptation of probiotics in the gut: selection pressures, optimization strategies, and regulatory challenges

**DOI:** 10.1038/s41522-025-00734-6

**Published:** 2025-06-07

**Authors:** Julia Leeflang, Josephine A. Wright, Daniel L. Worthley, M. Omar Din, Susan L. Woods

**Affiliations:** 1 Adelaide Medical School, University of Adelaide, Adelaide, SA 5000 Australia; 2 Precision Cancer Medicine Theme, South Australia Health and Medical Research Institute, Adelaide, SA 5000 Australia; 3 Colonoscopy Clinic, Brisbane, 4000 QLD Australia; 4 Department of Pediatrics, University of California, San Diego, La Jolla, CA 92093 USA

**Keywords:** Cellular microbiology, Evolution

## Abstract

Probiotics and live bacterial therapeutics are garnering increased attention for use in human health and have the potential to revolutionise the treatment of gastrointestinal diseases. However, a pervasive feature of bacteria that must be considered in the design of safe and effective probiotics and live bacterial therapeutics is their capacity for rapid evolution, both at the individual (epi)genetic level and in terms of population dynamics. Here we summarise gastrointestinal-specific evolution of bacteria, focussing on genetic and population levels of adaptation to factors such as carbon source availability, environmental stressors, and interactions with the native microbiome. We also address regulatory and safety considerations for the development of probiotics and live biotherapeutics from an evolutionary perspective, with a discussion of methods that utilise evolution to improve probiotic safety and efficacy via directed evolution, in comparison to another popular approach, genetic engineering.

## Bacterial evolution: a help or a hindrance?

Probiotics are defined as “live microorganisms which when administered in adequate amounts confer a health benefit on the host”^1^, whereas live biotherapeutics (LBP) are live microorganisms that are “applicable to the prevention, treatment, or cure of a disease or condition in human beings”^2^.

A seemingly neglected feature of both that requires significant consideration, especially in clinical contexts, is their rapid evolution. The combination of a high mutation rate, large population size, short generation time, rapid doubling times, and competence confers a high level of (epi)genetic adaptability to bacteria^3^. The consequences of such rapid evolution can be negative, resulting in, for example, pervasive antibiotic resistance, or positive, whereby strains can be optimised to a given environment in a short time frame. How evolution can be harnessed to promote desired traits of probiotics and LBP strains is an important point for consideration and is the focus of this review. We highlight the limited number of publications focussed on probiotic evolution within the host gastrointestinal tract (GIT), and even fewer for LBPs. As such, generalisations and inferences of bacterial behaviour based on studies investigating the gastrointestinal evolution of non-probiotic, exogenous and native bacteria are included to guide future research in this field.

## Mechanisms of bacterial evolution

**Fig. 1:**
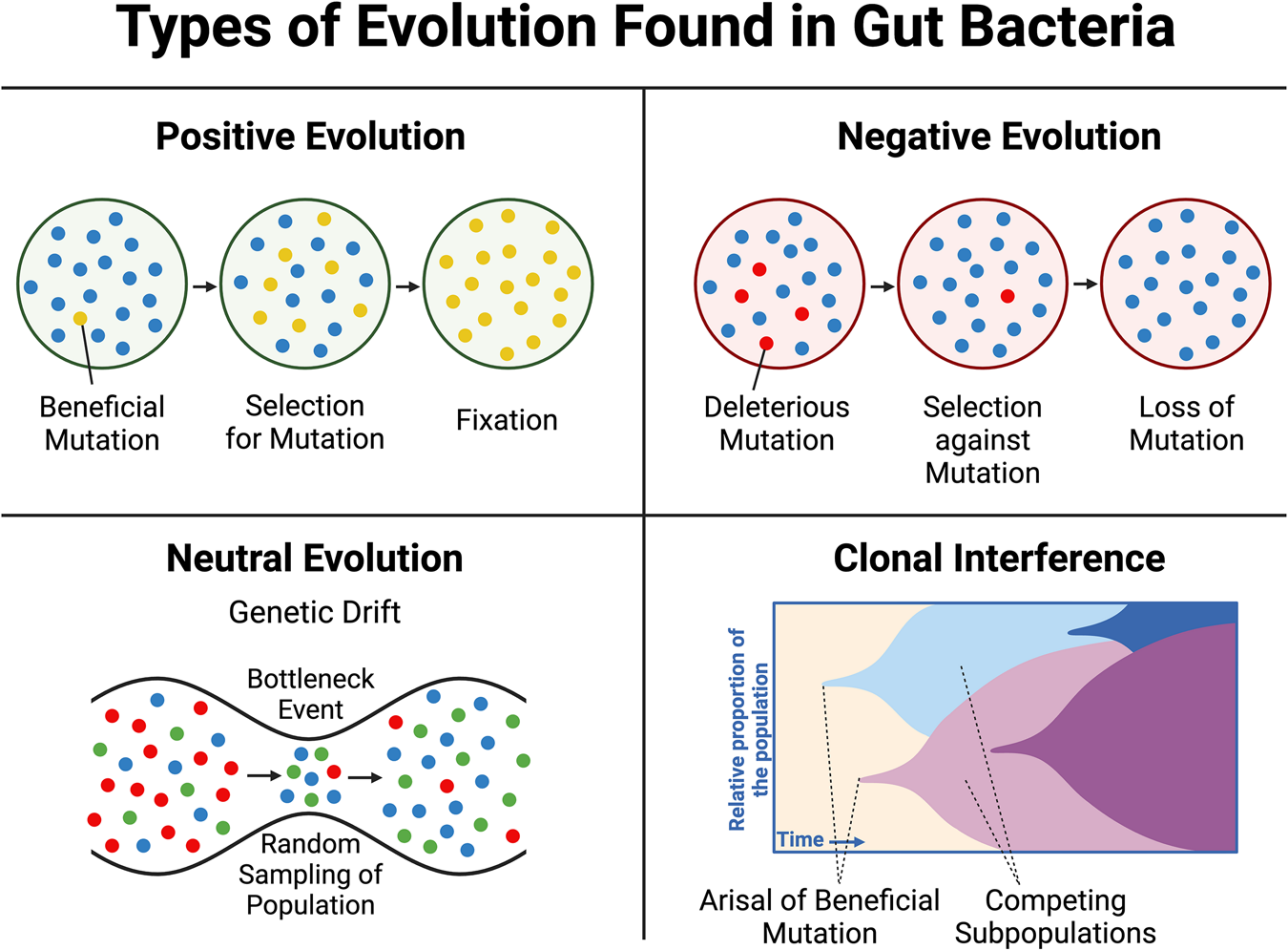
Types of evolution found in gut bacteria. Schematic of the main mechanisms of evolution found in gut bacteria. Evolution is driven by processes such as positive and negative selection of genetic mutations, neutral drift, and for asexually reproducing populations, such as bacteria, clonal interference. Created in BioRender.

The evolution of bacterial strains within the selective microenvironment of the gut has been assessed in detail in several hosts, utilising numerous strains. Bacterial adaption rate is greatest when initial fitness to a new environment is low^4,5^. The mechanistic basis of this adaptation encompasses a myriad of possible genetic- and population-level alterations (Fig. 1).

### Genetic Evolution

The most common forms of genetic alteration seen in bacteria within the gut environment are single nucleotide polymorphisms (SNPs) and indels (Box 1)^6,7^. In addition to these phenomena, insertion sequence (IS) transposition, horizontal gene transfer (HGT), and chromosomal restructuring are all observed sources of behavioural changes in bacteria (Box 1)^8^. These alterations can lead directly to loss-of-function or produce phenotypic gain-of-function via deactivation of repressor genes or increasing the copy number of a gene or its activator.

Adaptation to new niches requires the loss or downregulation of unprofitable phenotypes alongside the gain or upregulation of processes necessary for growth and survival in the new environment. For most species, this process is gradual, with changes taking years to centuries to occur. Bacteria, however, can evolve on incredibly short timescales of days to weeks. Standard bacterial DNA mutation rates usually range from 10^-7^-10^-9^ mutations per nucleotide per generation^9^. When considering the large population sizes that can double within minutes to hours, it is unsurprising that mutations can occur rapidly within bacterial populations. Although, these mutation rates can be elevated depending on the species or the environment. For instance, probiotic strain *E. coli* Nissle has an estimated mutation rate in the murine gut of 0.007 single nucleotide polymorphisms (SNPs) per generation per genome, allowing for extremely rapid evolution^10^.

Since greater mutation rates provide more opportunity for beneficial mutations to occur, it is unsurprising that elevating mutation rates increases colonisation ability, as shown in a study comparing DNA mismatch repair-deficient and wildtype *E. coli* K-12 colonisation to the germ-free (GF) mouse gut^11^. Some of the wildtype isolates in this study even naturally developed a hypermutator phenotype after monocolonisation, as assessed via spontaneous acquisition of rifampicin-resistance^11^. Hypermutator phenotypes have also been reported in probiotic *Lactiplantibacillus plantarum* HNU082 and *Bifidobacterium animalis* subsp. *lactis* V9 adapting to the mouse gut^12^, and in commensal *Bacteroides fragilis* within humans^13^. The benefit of hypermutation, however, is reduced once the population has adapted into its new niche, as extended hypermutation can lead to the accumulation of deleterious mutations that results in a reduced fitness in secondary environments, and this ability is therefore selected against and eventually lost^11,12^.

When examining genetic targets of mutation in non-probiotic *E*. coli K-12 after oral administration to the mouse gut^14–16^, and commensal *E*. coli ED1a within the human gut^6^, a common observation is modification to global regulator genes, which are responsible for the regulation of several genes of related function. These pleiotropic regulators are implicated as ‘genetic switches’ used to coordinate genes necessary for utilisation of different niches, thereby allowing for rapid adaptation to changing environments. As such, invading populations can rapidly adapt to the new environment by regulating phenotypes that would otherwise take numerous mutations to achieve, which is unlikely to occur before the population is lost from that environment. The pleiotropy of these genetic elements oftentimes results in a degradation of conserved traits when a mutation occurs at a site that also controls an adaptive trait under selection. As such, compensatory mutations are required to repair the loss of fitness, which often occurs quite rapidly^17^. This is most commonly seen when bacteria must evolve to cope with the costs of antibiotic resistance, where compensatory mutations have been found to occur at 10^-5^-10^-7^ per genome per generation in *E*. coli K-12 and *Salmonella typhimurium*^18,19^.

Box 1 Glossary **Bacterial epigenetics**

Epigenetics refers to any change to gene expression that is not the result of a change to the DNA sequence. In bacteria, this is largely controlled by DNA adenine methylation (as opposed to cytosine in eukaryotes).

**Single nucleotide polymorphism (SNPs)**

A single base pair change in DNA that, in protein-coding genes, can result in a change of a single amino acid. The effect on protein structure and function can vary between minimal to significant depending on the importance and nature of the amino acid affected. SNPs can also modify non-coding DNA such as gene regulatory regions.

**Insertion and deletion mutations (indels)**

The gain or loss of one or more bases in a gene. Indels often cause frameshift mutations that render proteins either partially or completely non-functional.

**Clonal interference (CI)**

The phenomenon where sub-populations of a strain with differing mutations of similar benefit compete for fixation. CI assumes an asexually reproducing population

**Insertion sequence (IS)** **Transposition**

Insertion sequences are mobile regions of DNA that can move within and between genomes of the same or different species. This is facilitated by transposon regions that flank the IS and are recognised by transposases encoded within the IS that cleave and integrate the IS in random locations in the genome.

**Horizontal gene transfer (HGT)**

HGT is the transfer of genetic material between cells. The mechanisms of HGT include conjugation (direct inter-cell transfer of genetic material), transformation (uptake of environmental DNA), and transduction (bacteriophage injection of DNA into the cell).

**Adaptive laboratory evolution (ALE)**

The process by which a microorganism is cultured in specific conditions for prolonged periods of time (typically weeks to years) to select for improved phenotypes, with no particular genetic target in mind. This may be preceded by genome-wide random mutagenesis.

**Directed evolution**

The process by which microorganisms with specific desired traits are created through iterative rounds of genetic diversification via targeted mutagenesis at the desired DNA region, and selection.

### Population evolution

A key mechanism of population evolution in bacteria is clonal interference (CI) (Box 1). In a slowly evolving population, such as a strain residing in its native environment, CI is less prevalent since a subpopulation with a beneficial mutation has time to dominate the population before the next occurs, thus resulting in a slow and sequential acquisition of mutations^20^. This domination of a single subpopulation is termed a hard selective sweep and results in a loss of genetic diversity as neutral and weakly beneficial or deleterious markers hitchhike with the beneficial mutation under selection. This low genetic diversity is detrimental to a population as it hinders adaption to changing conditions^20^.

In the case of faster evolution, such as an exogenous bacterium colonising a new environment, subpopulation competition is rampant; beneficial mutations develop faster than subpopulations can reach fixation. In this way, CI prevents hard selective sweeps, thereby retaining some genetic diversity. By extension, rapid mutation and a strong selection pressure can also lead to multiple clones within a population developing a different mutation for the same phenotype. Consequently, there are genetic soft sweeps that retain diversity whilst also producing an overall phenotypic hard sweep that can adapt the population to the given environment^15^. Due to the inherent competition involved in CI, it tends to favour fixation of stronger beneficial mutations. In this way, it has the effect of reducing weakly beneficial and deleterious mutations to near-neutral, a concept called emergent neutrality^21^.

CI can be observed in vivo experimentally, for instance, where mice are orally dosed with genetically identical *E. coli* K-12 differing only in neutral fluorescent genetic markers, and alterations to subpopulation size are assessed via fluorescent signals in stool isolates^15^. In the cases of a retention of equivalent fluorescent marker levels, whole genome re-sequencing revealed that adaptive mutations of equal strength had actually formed in each subpopulation, as opposed to the intuitive explanation of neutral evolution^15^. Conversely, another study investigating pathogenic *Salmonella* found significant enrichment of particular subpopulations and complete loss of most others after introduction of eight isogenic strains to the mouse GIT, as facilitated by acquisition of strongly advantageous mutations only in the enriched subpopulation^22^. While CI has not been explored experimentally for probiotics or LBPs colonising the GIT, this adaptative behaviour of bacteria in vivo is an important consideration for therapeutic dosing in people.

The concept of CI, while an important consideration, is not the dominating factor of bacterial evolution as it assumes a strictly asexually reproducing population. However, most bacterial species exhibit quasi-sexual selection, more commonly referred to as horizontal gene transfer (HGT), an important factor in biosafety assessments of any therapeutic strain in development. The mechanisms of HGT include conjugation (direct inter-cell transfer of genetic material), transformation (uptake of environmental DNA), and transduction (bacteriophage injection of DNA into the cell). Unlike the unwanted acquisition of antibiotic resistance, an interesting case of beneficial HGT in gut microbes was the discovery of genes responsible for digestion of seaweed-specific polysaccharides, such as porphyran and agarose, within gut commensal *Bacteroides* and *Firmicutes* in Japanese populations. These genes were naturally obtained from the genomes of marine Bacteroidetes, *Paenibacillus* and *Epulopiscium* spp. native to the seaweed, and their transfer was strongly selected for due to the prevalence of seaweed in the Japanese diet^23^.

The relationship between niche-specific selection pressures and bacteria can oftentimes be reciprocal. Certain stimuli can select for mutations that alter bacterial phenotypes such that the new behaviours change the surrounding environment. For instance, the pressure to catabolise a particularly abundant compound leads to the release of its substituent components, which can in turn exert their own selection pressures on the bacteria, or even different microbial species in a complex environment. This phenomenon is highlighted in findings of compulsory sequential mutation acquisition, where later mutations are only beneficial once the environment has been pre-conditioned by an earlier mutation developed in response to the initial environment^10,24^. This concept extends to the phenomenon of sub-population cross-feeding, where a beneficial gene may be most advantageous at a low-frequency such that the main fraction of the population can spare themselves the energy cost of expressing the gene, whilst feeding off the products of a sub-population forced to retain gene function to survive.

## Gastrointestinal selection pressures driving genetic evolution of gut bacteria

**Fig. 2:**
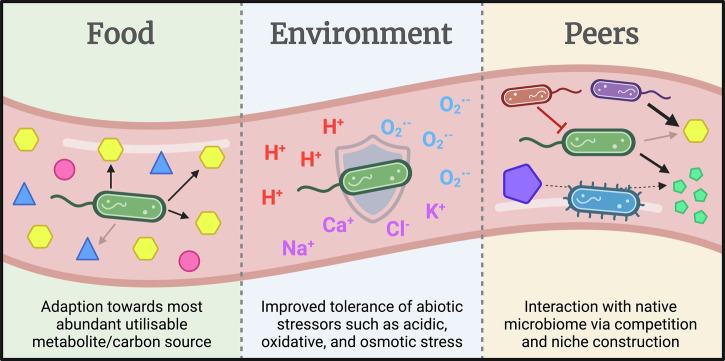
Gastrointestinal selection pressures on exogenous bacteria. Schema depicting the selection pressures exogenous bacteria adapt to within the gastrointestinal tract that include bacterial carbon source metabolism (Food), environmental stress tolerance (Environment), and interaction with the native microbiome (Peers). Created in BioRender.

Gastrointestinal bacteria experience many selection pressures within the GIT. The relative strength of a selection pressure can be inferred by the consistency and speed at which mutants for the process under selection arise. Basic survivability in a new environment is determined by the ability to utilise available nutrients and tolerate abiotic environmental stressors, so it is unsurprising that most mutations observed in bacteria introduced to the GIT are in relation to these processes (Fig. 2). Although some studies validate the effects of the discovered mutations, more needs to be done to explore the improvements to colonisation capabilities, and in the case of probiotics, functionality of gut-adapted lineages.

### Bacterial carbon source metabolism

Freter’s ‘nutrient-niche hypothesis’ postulates that a requirement of persistent colonisation of a microbe in a complex environment is the ability to utilise at least one limiting nutrient more efficiently than other species^25,26^. Consistent with this idea, many studies have found that gut evolution of bacteria, both native, and exogenous probiotic and non-probiotic strains, is dominated by alterations in carbon source metabolism, or ‘food’ (Fig. 2), as assessed by findings of mutational parallelism (i.e. same carbon source gene variant frequently mutated across isolates from multiple independent hosts), or increases in variant frequency beyond what is explainable by neutral drift (i.e. same carbon source genetic variant identified across multiple isolates within a single host).

For example, following administration of probiotic, non-probiotic or commensal *Escherichia coli* to various mouse models, genetic alterations are commonly reported at sites controlling metabolism, particularly involving transport, catabolism, and synthesis of amino acids^14,15,27^, carbohydrates^10,15,27^, organic acids^10,27,28^, alcohols^14,15,27^, and other general carbon source metabolism and respiration pathways^10,14,15,27^. These findings are not unique to *E. coli*; lactic-acid bacteria and *Bifidobacterium* spp., commensal strains commonly utilised as probiotics, also experience altered nutrient utilisation within the murine gut^12,29–35^. This highlights the necessity for a microbial species to rapidly optimise metabolism of available energy sources upon introduction to a novel environment.

In the human gut, similar findings of metabolism-based adaptation of bacteria have been observed^30,34,36–38^. For example, ingested probiotic *Lactobacillus plantarum* evolved mutations that enhanced rhamnose utilisation in both the mouse and human GIT, along with improved acid tolerance, which improved colonisation ability both in vitro and in vivo^30^. Additionally, parallel evolution between people of commensal non-probiotic *Bacteroides fragilis* populations was reported for mutations in genes involved in amino acid metabolism and encoding polysaccharide importers, along with cell-envelope biosynthesis, although these adaptations were not tested experimentally^13^. Comparison of current host-adapted strains of probiotic species to non-gastrointestinal strains can also elucidate historical co-evolution. A study comparing human-associated and free-living *Lactobacillus* strains found that the genes encoding glycoside hydrolases, that facilitate host-derived glycan metabolism, and other various carbohydrate metabolism and transport genes from human-associated *Lactobacillus* were absent in free-living strains, underlining the niche-specific necessity of these processes and importance of mimicking the in vivo environment during lab directed evolution or optimisation of new therapeutic strains^39^. Adaption to the gut environment can be very consistent across a range of host species; one study investigating candidate probiotic *L. plantarum* colonisation to human, mouse, and zebrafish guts found mutations for carbohydrate utilisation and acid tolerance within each host-adapted strain, which granted improved growth rates within in vitro assays^30^. Genomic analyses of already gut-adapted probiotics corroborate these conclusions, with early bifidobacteria and lactobacilli gut evolution resulting in the duplication of sugar metabolism and transport genes (reviewed in ref. ^40^).

Altered metabolic phenotypes reflect the composition of the environments to which they are adapting. For instance, NMR analysis of the GF mouse gut has revealed elevated levels of amino acids^27^ and specific sugars such as trisaccharide raffinose^41^. Consistent with this, mutant strains were found after passage of *E. coli* K-12 MG1655 through the GIT of these mice that had gained the ability to metabolise these carbon sources, which conferred greater fitness advantage when reintroduced to mice^27,41^. This shows the dependence of bacterial adaptation on the metabolic milieu of the environment, and how metabolomic analyses of target niches may guide optimisation of new biotherapeutic strains.

Host diet and microbial evolution are strongly associated, with more than 60% of gut microbial variation attributed to diet composition^42^. This strong influence of diet on the microbiome has been observed in several host types, including mice^10,43^, rats^44,45^, humans^46,47^, zebrafish^48^, and fruit flies^49^. Furthermore, race-specific human microbiome compositions are largely attributed to dietary differences^46,50^. Together, these studies show that colonising bacteria preference the most abundant carbon source at their disposal, the identity of which is largely influenced by host diet.

### Environmental stress tolerance

The GIT is a harsh environment. As such, stress tolerance to abiotic factors, or ‘environment’ (Fig. 2), has been shown to play an important role in influencing bacterial adaptation. For instance, the hyperosmotic environment of the gut, produced by high solute concentrations, and the presence of reactive oxygen species (ROS) are strong stressors on invading bacteria. Therefore, improved osmotic and oxidative tolerance are favourable mutations found in both non-probiotic *E. coli* K-12, MG1655^16,27,51^ and probiotic *Lactobacillus helveticus* MTCC 5463^52^. Interestingly, osmotic tolerance can be achieved via a reduction in permeability through repression of porin expression^16^, and overlap between osmotic and oxidative stress tolerance has also been found in *E. coli* K-12 MG1655, as lactose-induced osmotic stress led to the upregulation of the oxyR regulon, previously implicated in oxidative stress tolerance^51^. Unsurprisingly, acid tolerance is also consistently reported in probiotic bacteria post gut transit, including *E. coli* Nissle^10^, *Lactiplantibacillus plantarum* HNU082^12,30^, and *Lactobacillus kefiranofaciens* ZW3^53^, which provides improved colonisation ability in response to the notoriously low pH of the GIT.

The significance of environmental stress as a selection pressure is highlighted in findings of increased evolution of non-probiotic *E. coli* strain K-12 in the guts of older mice. Aging is associated with higher inflammation, producing stronger selection pressures for stress tolerance in invading bacteria. Although greater evolutionary change is seen overall, these bacteria exhibit slower adaption towards altered metabolism than their young mouse gut-adapted counterparts^14^.

Another factor indicating the importance of environmental stress tolerance is the fact that multiple studies have found that the first mutational step of non-probiotic *E. coli* strain K-12 MG1655 adaption to the mouse gut is inactivation of the *gat* operon, which controls galactitol metabolism^14,15,24,27,54^. Galactitol analogues can have an inhibitory effect on *E. coli* and are present in the gut as the reduction product of galactose metabolism^27,51^. Although a shining example of interhost convergent evolution, the applicability to other bacterial strains is likely quite low as *E. coli* K-12 MG1655 is unique in that it has an IS insertion in the *gat* repressor, resulting in constitutive activation of the operon^28^.

### Interaction with the native microbiome

In exploring the intricate dynamics of evolution of bacteria within the gut, the strong influence of the native microbiome, or ‘peers’ (Fig. 2), must be considered. There are several different forms of interaction between microbes. These ecological relationships can be competitive, predatory or symbiotic. Symbiotic relationships are where one species benefits and the other either also benefits (mutualism), is unaffected (commensalism), or is at a loss (parasitism).

Each relationship is important for gastrointestinal bacterial evolution, but each has its own significance in different contexts. The nutrient niche hypothesis implies that the introduction of more adapted species would further limit niche availability through direct competition for resources and attachment sites, consequently reducing opportunities for beneficial mutations. Conversely, an alternative perspective, encapsulated in the hypothesis of ‘diversity begets diversity’, contends that greater microbiome diversity fosters increased opportunities for evolution through symbiotic inter-species interactions such as niche construction^55^, for example one species cross-feeding on the products of the degradation of larger metabolites by other members in the community, which is supported by multiple studies^12,27,31,33,54^.

This viewpoint is at odds with the previously cited findings of a reduction in evolution of both lab- and gut-adapted *E. coli* strains, K-12 (non-probiotic), and Nissle (probiotic), respectively, in the presence of greater microbiome diversity in the mouse gut^10,27^. However, a study comparing the evolution of the probiotics *Lactiplantibacillus plantarum* HNU082 (Lp082) and *Bifidobacterium animalis* subsp. *lactis* V9 (BV9) within both GF and specific pathogen-free (SPF) mouse models found that adaption was greatest in the more diverse microbiome^12^. 100–1000-fold more mutations were found in each strain after passage through the SPF mice, suggesting that resource competition between microbes is a stronger selection pressure for these probiotics than host factors^12^. Discrepancies between each study can likely be attributed to the bacterial strains utilised, as the same selection pressures can act differently on bacteria with varied genetic backgrounds and initial fitness; *E. coli* may be naturally more adapted to environments pre-conditioned by the interactions of other microbes rather than the GF gut, which has stark physiological differences to a conventional GIT with a complete microbiome^10^. This also explains the within-study differences between Lp082 and BV9, where the faeces-derived BV9 exhibited less mutation than Lp082, which was isolated from food products, likely because its initial state was more fit for the gut environment^34^.

Bacterial species fill the best niche available to them, making them sensitive to competing microbiota capable of filling the same niche. In a study investigating bi-colonisation of the GF mouse gut with non-probiotic, *E. coli* K-12 strain and the competing species, *Blautia cocoides*, they found *E. coli* evolution shifted away from metabolism of amino acids, as seen in mono-colonised mice, to that of organic acids, along with selecting for mutations in fumarate/succinate transport and anaerobic respiration. Interestingly, the adaptive loci identified in the co-colonisation model were more analogous to the loci found in *E. coli* after colonisation of antibiotic-treated SPF mice, with a diverse anaerobic microbiome, than GF mice^27^.

An interesting example of microbiome influence on bacterial adaption is the finding of variable exogenous bacterial adaption between immuno-competent and -deficient mice, where the latter saw slowed and more variable adaption of colonising, non-probiotic *E. coli* K-12 strain. Remarkably, when the microbiomes of each mouse type were homogenised, the difference in evolution between hosts was lost, implicating the dysregulated microbiota in immunocompromised mice as the source of the altered adaption dynamics^54^.

### Secondary processes driving bacterial evolution in the gut

Once the key issues of nutrition and environmental stress tolerance have been overcome, secondary processes, such as adherence to host epithelial cells^27^, ribosomal RNA maturation^28^, immune hiding^16^, and commonly, motility^16,27,41,56^, can be enriched in the gut microenvironment, often in a diet-, host- and strain-specific manner. For example, the alterations in motility seen within *E*. coli K-12 are usually via repression of flagellin expression^16,27,41,56^. Possible explanations are that flagella are a common pathogen-associated molecular pattern that trigger an inflammatory response and lead to immune clearance of the invading bacteria^16,57^. Therefore, eliminating this trigger may allow for persistence of the strain within the gut. Alternatively, the direct energy cost of synthesis and rotation may be better suited to other processes^56^. The *flhDC* operon responsible for this motility phenotype is also implicated in the regulation of citrate synthase, succinate and malate dehydrogenases, and galactose transporters^56^. Therefore, derepression of these systems may also lead to an improved level of carbon metabolism^56,58^.

## Harnessing evolution

To limit the risk associated with the evolution of probiotics/LBPs in humans, prior adaptation of new strains to the host environment using animal or in vitro model systems may be beneficial and enable assessment of the performance and safety of new strains prior to administration to humans^12,59^. This can be achieved with adaptive laboratory evolution (ALE) (Box 1), which can incorporate relevant environmental stressors and modelling with host microbiota as reported^60^, which is also likely to improve engraftment and efficacy.

**Fig. 3:**
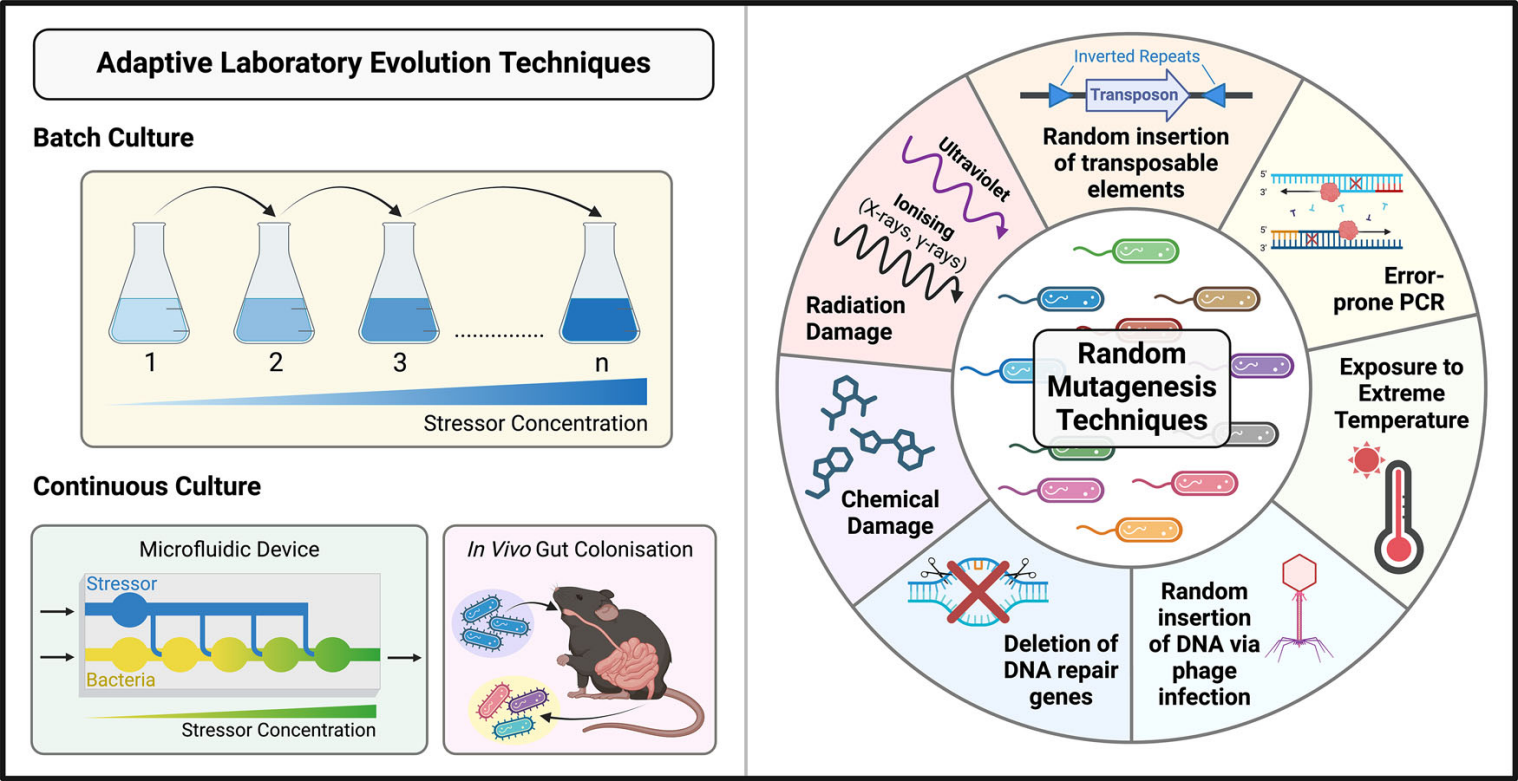
Adaptive laboratory evolution and random mutagenesis techniques. Adaptive laboratory evolution (ALE) techniques include batch culture, where a microorganism is seeded into cultures of increasing stressor concentration, or continuous culture under certain conditions such as within microfluidic devices or chemostats in vitro, or in vivo within a biological system such as a murine model. ALE may be preceded by random mutagenesis to increase genetic variability available to selection, undertaken using a variety of techniques. Created in BioRender.

Beyond mitigating safety concerns, ALE can be a powerful tool in improving probiotic/LBP survivability and beneficial behaviour. There are multiple techniques used to improve particular bacterial functions (Fig. 3). A simple method is repeated culturing under a certain stressor to evolve tolerance. For instance, sequential culturing through increasingly oxidative conditions produced a strain of the obligate anaerobe and candidate probiotic, *Faecalibacterium prausnitzii* DSM, with notable oxygen tolerance^61^. This concept has been expanded to more novel technologies such as microfluidics, where the probiotic *Lacticaseibacillus rhamnosus* GG was exposed to increasing hydrogen peroxide concentration through a microfluidic device and thereby developed better capacity to grow under oxidative stress^60^. A similar concept is repeated dosing of a particular host to improve probiotic adaption to the gastrointestinal system, such as a study on the probiotic *Lactobacillus plantarum* WCFS1 showing increased digestive tract retention time after three passages through the mouse gut^62^. Coupling ALE with random mutagenesis introduces more genetic variability and increases the avenues for selection, providing a greater chance of the desired trait being achieved. There are multiple methods to achieve such a mutant library (Fig. 3), the most common include physical (UV or ionising radiation) or chemical mutagenesis, with each choice providing different types of mutations and levels of mutational burden. Similarly, directed evolution (Box 1) can be employed if there is a known genetic target that can be mutated and screened for improvements in that particular phenotype. In contrast, limiting natural evolution of probiotic or LBP strains can improve unintended evolution in the host. Strategies to limit this can include deletion of error-prone DNA polymerases^63^, or upregulation of DNA repair proteins^64^.

Alternately, specific characteristics can be rationally designed into probiotics/LBPs through targeted genetic modification of the bacterial chassis organism. This has the advantage in this age of genomic sequencing and synthetic biology advances of being relatively fast in comparison to evolutionary approaches, utilising prior knowledge, and being specific for a desired behaviour. There have been many preclinical successes utilising GMO probiotics, including use in infection, inflammation, metabolic disorders, and cancer treatment and diagnosis (examples reviewed in ref. ^65^), and the field is beginning to progress into clinical trials for treatment of various metabolic and gastrointestinal disorders, and many cancer types (reviewed in refs. ^66,67^). Genetic engineering encounters regulatory difficulties associated with GMOs that are not relevant to naturally evolved genetic changes. Genetic modification can generate unforeseen consequences and so requires careful consideration of design and testing. Genetic modifications should be designed to limit genetic instability and potentially harmful components^68^. These considerations include the removal of antibiotic resistance cassettes, plasmid-based genetic constructs should use stabilisation systems or be integrated into the genome and introduction of coding genes should be limited to those genes already found in the host microbiome, rather than introducing new genetic elements to the ecosystem.

## Regulatory and Safety Considerations

A core tenet of the international guidelines for development of new therapeutics (International Council for Harmonization of Technical Requirements for Pharmaceuticals for Human Use, ICH) is that the product provide therapeutic benefit that outweighs potential risk. In keeping with these guidelines, the isolation and characterisation of novel probiotic strains typically includes consideration of epidemiological association with ‘health’ rather than ‘disease’ and complete genomic and functional analyses to select non-toxic strains without known pathogenicity or antibiotic resistance genes/traits^69^. Good manufacturing practices (GMP) are used to produce probiotics and LBP products for human consumption, but GMP stops at the generation, quality and stability of the final product prior to use^70^. We posit that analysis of potential safety concerns should include analysis of bacterial adaptation to human hosts following administration, particularly in the intended target population in combination with standard care treatment modalities. Biosafety concerns resulting from (epi)genetic evolution of probiotics and LBPs primarily arise from the potential for HGT between the introduced strain(s) and the commensal microbiome, that may result in unwanted traits being transferred to the introduced strain or host microbiota^66^.

An important point to consider when comparing the relative risk involved with genetic engineering to ALE of probiotics or LBP to enhance specific traits, is the introduction of non-native genetic information inherent to the production of genetically modified probiotics or LBP. This can increase risk by introducing additional genetic sequences that can introduce unintended metabolic shifts, trigger an aberrant immune response, or be transferred to the native microbiome. This could include transfer of pathogenic gene islands or uptake of antibiotic resistance, as has been observed in preclinical studies^71,72^. Interestingly, transfer of naturally occurring tetracycline resistance from probiotic *Lactobacillus reuteri* to human gastrointestinal microbiota could not be demonstrated in a mouse study^73^, however a genetically modified strain containing an exogenous tetracycline resistance gene was not included as a comparator in the study to see if addition of exogenous genetic sequence to the probiotic strain increases risk of transference to the host microbiome.

Equally, alterations to gut microbiota composition or relative abundance of taxa have been associated with disease and may result unpredictably from adaptation and colonisation by an exogenous LBP. Generally, however, gross modifications to the host gut microbiome following supplementation with a biotherapeutic strain(s) are difficult to detect without using targeted approaches, such as a PCR assay specific to the strain of interest. While these processes of genetic mixing and modifiable population composition are naturally occurring phenomena inherent to any complex microbiota, the adaptive process may be accelerated upon introduction of an exogenous bacterium to a new environment. Biotherapeutic strains are rarely re-isolated from people post-administration to assess (epi)genetic evolution of the strain. We would encourage such analyses as they may provide a rich source of information on mechanisms of host adaptation specific to the LBP and prove fruitful to improve bacterial chassis safety and/or viability and efficacy in the future. Here we focussed on bacterial adaptation to the gut environment but equally these considerations are important for development of probiotics/LBPs for use in alternate body sites such as the skin^74^, vagina^75^, respiratory tract^76^, or otolaryngological cavities^77^. An interesting example of the latter is recent preclinical advances in use of GMO-bacteria to deliver therapeutics across the blood brain barrier following intra-nasal administration in a mouse model of obesity^78^.

## Summary

In the development of new probiotic and LBP strains, consideration must be given to the natural evolution for which bacteria are notorious. We can learn from the knowledge base of bacterial adaptation reported in the gastrointestinal tract to inform this development. GIT-specific evolution is characterised by adaption to the best available carbon source, which is largely dependent on host diet and existing microbiome composition, and also increased tolerance to stressors such as osmotic and oxidative stress and acidity. Several factors can influence the evolution of exogenous bacteria in the GIT, such as host diet and native microbiome composition. Optimising probiotics or LBPs to the GIT has the potential to significantly increase their efficacy and also allows for in-depth assessments of stability and adaptation important to address potential safety concerns. This can be achieved with animal or in vitro models that encompass as many GIT-specific characteristics as possible. In the case of disease treatment, these models should also be tailored to the target disease state to generate disease-specific adaptative genetic alterations, important for efficacy and biosafety analyses of the LBP.

## Data Availability

Data sharing is not applicable to this article as no datasets were generated or analysed during the current study.
